# Welfare Status of Working Horses and Owners′ Perceptions of Their Animals

**DOI:** 10.3390/ani7080056

**Published:** 2017-08-01

**Authors:** Daniela Luna, Rodrigo A. Vásquez, Manuel Rojas, Tamara A. Tadich

**Affiliations:** 1 Programa Doctorado en Ciencias Silvoagropecuarias y Veterinarias, Universidad de Chile, Santa Rosa 11315, La Pintana, Santiago 8820000, Chile; danluna@veterinaria.uchile.cl; 2 Instituto de Ecología y Biodiversidad, Departamento de Ciencias Ecológicas, Facultad de Ciencias, Universidad de Chile, Las Palmeras 3425, Ñuñoa, Santiago 7800003, Chile; rvasquez@uchile.cl; 3 Departamento de Ingenieria Industrial, Facultad de Ciencias Físicas y Matemáticas, Universidad de Chile, Beauchef 851, Santiago 8370456, Chile; manuelrojas@uchile.cl; 4 Departamento de Fomento de la Producción Animal, Facultad de Ciencias Veterinarias y Pecuarias, Universidad de Chile, Santa Rosa 11735, La Pintana, Santiago 8820000, Chile

**Keywords:** equine welfare, working horses, urban draught horses, semantic analysis, human-animal relationship

## Abstract

**Simple Summary:**

Appropriate strategies aimed at improving the welfare of working horses should contemplate the assessment of welfare status, as well as the evaluation of the human–animal relationship within each geo-cultural context. We assessed and compared the welfare status of working horses in two administrative regions of Chile and explored the nature of the owner–horse relationship from the perspective of the owner. The overall prevalence of health problems and negative behavior responses was low. However, significant differences between regions exist in the presence of lesions and the person responsible for managing horseshoeing. Two differing views were found regarding the owners’ perception of their horse: predominantly affective or instrumental. Despite the instrumental perception predominantly residing in one region, the affective perception was widely shared by owners in each region. The findings suggest that Chilean working horses have a, generally, good welfare and that the development of an affective owner–horse relationship is possible. Additionally, the results suggest that affective and instrumental perceptions of these animals can coexist.

**Abstract:**

Appropriate interventions to improve working equine welfare should be proposed according to scientific evidence that arises from different geo-cultural contexts. This study aims to assess and compare the welfare status of working horses in two administrative regions of Chile and to determine how owners perceive their horses. Horses’ welfare status was assessed through direct indicators (direct observation and clinical examination) and indirect indicators (an interview with the owner). Owners′ perceptions of their horses were determined through a discourse analysis of their statements. In total, 100 horses and 100 owners were assessed. Results showed a low prevalence of health problems and negative behavior responses among horses in the two regions evaluated. Significant associations were found between inadequate body condition and the absence of deworming, and between hoof abnormalities and a low frequency of shoeing. Between regions, significant differences were found in the presence of lesions and the person responsible for horseshoeing. In regards to the owners′ appreciations, two differing perceptions of working horses were found: a predominantly affective perception and a perception of the animal as a working instrument. Although the instrumental perception was more frequent in the Araucania region, the affective perception was widely shared by both owner populations. The results reveal a good welfare status in working horses and suggest that both affective and instrumental perceptions of these animals can coexist.

## 1. Introduction

In developing countries such as Chile [1], working animals provide an essential resource of power for millions of people who live in poverty [2,3]. In the case of working equids, there is increasing evidence of their socioeconomic contribution to human livelihood through their direct and indirect impact in generating income for thousands of households worldwide [3,4]. It has been reported that the welfare state of these equids is usually poor and impacts directly on their health, mental state and working capacity [5,6,7]. This may seriously compromise the well-being of these animals and the families they work for. For this reason, the World Organization for Animal Health (OIE) recently decided to develop the first welfare standards for working equids used for traction, transport and income generation [8]. However, it is important to highlight that welfare problems, husbandry practices, and the role that these working animals play can vary between countries, through time, and even within the same community or locality [5,6]. Consequently, appropriate intervention strategies to improve the welfare of these animals should be proposed and implemented according to, at least, two criteria: the main welfare problems found in the different geo-cultural contexts, and the assessment of the quality of the human–animal relationship [5].

The welfare status of working horses in Chile has been previously assessed [9,10,11], but the influence of geographic and cultural differences on these animals′ welfare is unknown. Differences within a country could modify the risk factors associated with the animals′ welfare. For example, heat stress and dehydration are conditions that negatively affect the welfare of equids in countries with arid climates, such as India or Pakistan, where ambient temperatures of up to 48 °C are found [5]. However, these problems have not been observed in countries that have internally varying climatic and geographic conditions, such as Chile. For example, the Metropolitana de Santiago region has the largest urban population in the country and is characterized by a warm, temperate climate with a prolonged dry season [12]. In comparison, the Araucania region is one of the poorest regions and has the highest percentage of rural population. It is characterized by four types of climate, predominantly a rainy, temperate climate [13]. Moreover, this region has the largest number of individuals belonging to the Mapuche group, an indigenous, ethnic population that preserves their ancient traditions [14].

Strategies oriented to improve the welfare of animals, their owners and caretakers, also require an appropriate understanding of the human–animal relationship and the multiple factors that modulate it [15]. To form these strategies, the motivational considerations (bases) that underlie attitudes towards animals must be identified [16], primarily the emotional or instrumental ways in which people relate to animals [17,18]. Attitudes toward animals are often the focus of human–animal interaction studies [19,20,21,22] and, more recently, of welfare studies [23]. However, most research on human–horse relationships and the perceptions of horses has been centered in the equestrian world (horses that are kept primarily for recreational riding or competition) [18,24,25,26] and there is little information on working equids despite the important implications that this knowledge holds for improving these animals′ welfare. Moreover, welfare studies on working horses have not addressed the importance of owner′s attitudes and perceptions on their interactions with their animals [4,5,6,7,9]. 

Most studies that focus on the perception of horses have been limited to the use of traditional qualitative discourse analysis to determine the diverse perceptions and conceptions that people have in relation to these animals [18,25,26,27]. None of these studies have explored the representation of the meaning of words from other perspectives, such as Latent Semantic Analysis (LSA). LSA is a mathematical tool that has been proposed by psychology researchers as a method for extracting and representing the meaning of words [28] obtained in written texts, interviews or free text surveys [29,30]. In contrast to traditional discourse analysis, LSA is an interesting tool for inferring much deeper relationships between words and results in better predictions of human judgments [28]. Determining, through LSA, how owners or caregivers perceive their animals could reflect the nature of the owner–horse interaction and the animals’ role in their lives. It could also infer the owner’s motivation for improving the well-being of their horse.

Taking this into account, the aims of this study were, firstly, to assess and compare the welfare status of working horses in two different administrative regions of the country: one of which is closer to an ethnic-rural background (Araucania region) than the other (Metropolitana de Santiago region), and to determine whether any associations between direct and indirect welfare indicators exist. Secondly, we aimed to determine owners′ perceptions of their horses in both regions. 

## 2. Materials and Methods 

This study was carried out in peri-urban neighborhoods in two regions of Chile: the Metropolitana de Santiago region (33°26′16″S 70°39′01″O) and the Araucania region (38°54′00″S 72°40′00″O), between March of 2015 and January of 2016. The welfare assessment protocol was approved by the Animal Use and Care Ethical Committee of the School of Veterinary Sciences of the University of Chile (N° 06-2015). Owner participation was voluntary after signing an informed consent agreeing to participate in the study under the understanding that no economic benefit was involved. The owners were informed of all the aims of the study. 

Due to a lack of information on the number of working horses in Chile, a convenience sample size was used. To localize owners and their horses, an electronic consultation with local municipalities was held to geographically locate the cities in Chile where a significant number of these horses were currently working. The researchers then visited the residences of the owners to invite them to participate in this study.

### 2.1. Welfare Assessment Protocol for Working Equines

A total of 100 urban draught horses (48 from the Metropolitana de Santiago region and 52 from the Araucania region) were assessed using a welfare assessment protocol for working equids, based on previously published literature [4,5,9,31]. When the owner had more than one horse, one horse per owner was randomly selected for the analysis. This protocol included a set of direct indicators, such as health parameters and behavioral observations, for assessing the general welfare status of horses (Table 1 and Table 2). In addition, indirect indicators, such as resource-based measures (the provision of food and water, management practices, etc.) were included (Table 3). Additional information about the general characteristics of the horses, such as age, sex, conformation type and estimated live weight, were also recorded. These indicators were evaluated at the owners’ households. First, the behavioral assessments were performed through direct observation. The health parameters were then assessed through clinical examination (direct indicators). Finally, each horse owner answered a specific questionnaire to obtain general characteristics of their horse, and the main resources and management that they received (indirect indicators). 

#### 2.1.1. Direct Welfare Indicators

An assessment of horses’ health status and behavioral parameters was performed by an observer (a veterinarian) using a standardized protocol (Table 1 and Table 2). The presence and location of skin lesions were recorded, including lesions at the labial commissures of the mouth. A Body Condition Score (BCS) was recorded for each horse using a standard scoring scale from 1 (emaciated) to 5 (obese) [32], including half scores [6]. The assessment of hoof shape, conformation and quality was based on the criteria of Popescu et al. [33] and Burn et al. [31] (see Table 1). Hair coat and skin condition, including the presence of external ectoparasites, were assessed based on the criteria of Pritchard et al. [5]. Gait abnormalities (lameness) were assessed and recorded by observing a horse walking in a straight line for approximately 20 m. The presence of lesions, hoof abnormalities, coat and skin conditions, and gait abnormalities were recorded as either present or absent, and adequate or inadequate, in terms of whether an indicator was altered or within the normal range. Body condition was recorded as a score and either adequate or inadequate (scores of 3, 3.5 and 4 were considered adequate, whereas scores of 1, 1.5, 2, 2.5, 4.5 and 5 were considered inadequate). The health parameters were also assessed in the same process as described previously.

Observations of horses′ general attitudes (alert, apathetic or depressed), horses′ responses to the approach of both the observer and the owner, horses′ responses to the observer and the owner walking down the animal′s side (“walk-by”), and horse′s responses to chin contact by the observer and the owner were made similarly, in accordance to the welfare assessment protocol as described by Popescu and Diugan [4] and Burn et al. [31] (see Table 2). In the approach and “walk-by tests”, each horse’s response toward the observer and the owner was categorized as indifference, friendliness, avoidance or aggressiveness, based on the criteria of Popescu and Diugan [4] (see Table 2). The horse′s response in the “chin contact test” was categorized as avoidance or acceptance based on the criteria of Burn et al. [31] (Table 2). Finally, the “picking up a limb test” was also included in this study. Each horse′s response towards the owner and the observer, when they picked up a limb, was recorded as either avoidance or acceptance. This was done in order to assess the response towards a common handling routine (cleaning of the hoof and shoeing). The behavioral tests were made first by the observer and then by the owner. Each horse′s response was observed and recorded by the observer. The only procedure not made by the owner was that of general attitude (Table 2).

#### 2.1.2. Indirect Welfare Indicators 

A total of 100 urban working horse owners (*n =* 48 from the Metropolitana de Santiago region and *n* = 52 from the Araucania region), most of them men (*n* = 90) ranging in age from 17 to 83 years (average = 43; SD = 15.03), participated in this study. Each owner was interviewed using a standardized, structured questionnaire, which included a combination of open and closed questions to register information about feeding practices (frequency of feeding, water availability), working practices (work type and frequency), horse shoeing practices (frequency), preventive managements (deworming), and veterinary consultation (Table 3).

#### 2.1.3. Horses′ General Characteristics

(a) Age, determined by the horse’s history as recounted by the owner and confirmed by the inspection of teeth; (b) Sex, recorded by observing the external genitalia; (c) Anamorphosic Index (AI), calculated to establish whether the horse had a speed or draught type morphology, based on the equation and criteria described by Cassai [34]: (HG)^2^/HW, where HG is the heart girth and HW is the height to the withers, expressed in meters. The equine is considered to be of draught type if the AI is greater than 2.12, and a speed type if it is lower. (d) Estimated live weight, using the modified equation for Chilean horses described by Meyer [35]: HG^2^ x EIL/11.462, where HG is the heart girth and EIL the shoulder-*tuber ischii* length, expressed in centimeters.

### 2.2. Owners’ Perception of Their Horses 

After recording the direct and indirect welfare indicators, owners were asked to answer the following open question: “What does your working horse mean or represent for you?”. Owners with more than one horse were asked to answer for the totality of their horses. This broad question gave owners the opportunity to express, in their own words, their subjective perception and point of view about how they conceive and conceptualize their working horse. Such an approximation has been used in other studies, such as Birke [26], Birke et al. [25] and Shuurman [18]. The owners′ responses were recorded and transcribed verbatim by the researcher.

### 2.3. Statistical Analysis

The data from 100 horses and 100 owners was incorporated and stored in an Excel spreadsheet (Microsoft Office Excel^®^ 2013) and then exported to SPSS (IBM version 22.0.0.0 for Windows, Armonk, NY, USA) for further statistical and graphical analysis. Descriptive statistics (means, standard deviation and percentages) were used to summarize the information on the general characteristics and welfare state of horses for each location. 

The Wilcoxon rank sum test and the Student’s t-test were applied to determine significant differences between regions for the variables: age, estimated live weight, AI, BCS, feeding frequency, work and shoeing frequency. The association between the frequency of each indicator and location, as well as interactions between animal-based and resource-based information, were examined using the Chi-squared test and Fisher′s exact test. A statistical significance level of *p* < 0.05 was established.

In order to determine the differences in the conceptions and interpretations of working horses embedded in the statements of owners, a linguistic analysis of each owner’s discourse, through text analytics (TA), was applied. These analyses included cluster analysis, correlations of terms (Spearman′s correlation) and Latent Semantic Analysis (LSA). 

Latent Semantic Analysis is a natural language processing technique developed by Landauer and Dumais [36]. The technique allows for a mathematical representation of the relationship of meaning between words and sentences contained in a written text. Initially developed for library indexing, it is now applied to the analysis of quantitative literature reviews, textual data in computer-mediated communication, interviews, and management of knowledge repositories [29]. This is all possible through the statistical techniques that identify relationships between sentences in a collection of documents, thus generating a specialized domain of evaluation that allows an analysis to be performed.

The LSA was used to identify the degree of proximity of words within the semantic space evaluated. In this study, the LSA was applied in conjunction with cosine measurement to determine the contextual meaning and latent relationships behind the words of owners, specifically to determine concepts associated with the term “horse” in each region.

In the determination and implementation of the different semantic algorithms of this study, the following tools and software-type product-license and versions were used: (a) System Operative Linux Debian-Ubuntu, GPL-GNU (General Public License v3.0), kernel 3.13.0-35-generic; (a: Linux open source, New York, USA) (b) R-CRAN, Cluster of library of analysis and modeling, v3.1.1; (c) IDE RSTUDIO, Integrated Developmental Environment-GNU, v.098.1028; (d) Package tm, Library R-CRAN-GNU, v0.6-2; (e) Package lsa, Library R-CRAN-GNU, v0.6-2; (f) Package ggplot2, Library R-CRAN-GNU, v1.0.0; (g) Package igraph, Library R-CRAN-GNU, v0.7.0. (b–g: The R Project, open source, The R Foundation, Vienna, Austria)

The Unix-based operating system, and particularly Linux, added to the tools used which are supported under the GLP-GNU licenses-General Public License V3.0-features that allow them to be used to study, share and copy, as well as modifying the software. This environment allows an open implementation and the ability to make the modifications and adjustments necessary to achieve the integration of different tools.

## 3. Results

### 3.1. General Characteristics 

The average age of the study population of working horses was 8.7 years (range = 2–25; SD = 4.5 years). Sixty-one percent of horses were mares, 29% geldings, and 10% stallions (Table 4). Most horses (70%) had a speed type conformation according to their anamorphosic index and the average estimated live weight was 413 kg (range = 185–632; SD = 82 kg). General characteristics of horses within each region are shown in Table 4. Between regions, no significant differences were found in relation to age (Wilcoxon rank sum test, *p* = 0.572), estimated live weight (Student′s *t*–test, *p* = 0.315), and the percentage of horses with draught conformation (X² = 2.21; *p* = 0.190). However, there was a higher frequency of geldings in the Araucania region, compared to the Metropolitana de Santiago region (Fisher′s exact test, *p* = 0.001), which, in turn, presented a higher frequency of stallions (Fisher′s exact test, *p* = 0.006).

### 3.2. Working Horse Welfare Assessment: Direct and Indirect Indicators

The summarized results of health indicators and behavioral parameters assessed in working horses in each region are shown in Table 5 and Table 6, respectively. Significant differences between regions are also indicated.

The evaluation of health indicators in the total population showed that most horses (83%) had an adequate body condition score (average = 3.3; SD = 0.56; range = 2–5). No significant differences (Wilcoxon rank sum test, *p* = 0.08) were found between regions in relation to the BCS of the horses. The main welfare problems found were hoof abnormalities (53%) and the presence of skin lesions (47%), which were mostly simple excoriations located on harness-related areas. Other less frequent problems found were inadequate skin or coat condition (14%), limb-associated abnormalities such as gait abnormality and lameness (13%), and lesions at the labial commissures of the mouth (3%). Significant differences between regions were found only in the presence of lesions: horses from the Metropolitana de Santiago region had a significantly higher (X^2^ = 4.7; *p* = 0.03) frequency of skin lesions, primarily in the head and neck area (Fisher’s exact test, *p* = 0.0003).

The prevalence of behavioral responses displayed by the horses toward their owner and the observer for the five behavioral tests applied are shown in Table 6. Most horses had an alert attitude (97%) and presented positive (friendly) responses towards both the owner and the observer. No significant differences (*p* > 0.05) were found in a horse′s responses towards their owner and the observer in the four behavioral tests. Regarding the differences between regions, there was a significantly higher (*p* < 0.01) frequency of horses with friendly responses toward both the observer and the owner in the approach test and the walk-by test in the Araucania region in comparison to the Metropolitana de Santiago region (see Table 6). Aggressiveness, avoidance and indifference were the least frequent reactions in all tests, with no significant differences between regions (*p* > 0.05) (Table 6).

No significant difference (Wilcoxon rank sum test, *p* = 0.15) was found between the two regions regarding feeding frequency. The availability of water had no significant association (Fisher′s exact test, *p* = 0.18) with the horses′ regions of origin. Most horses (83%) were fed twice or three times per day and almost all of them (90%) had access to drinking water throughout the entire day, as declared by the owner. Regarding equine healthcare, approximately half of the horses (49%) had been dewormed within the last 6 months. However, 17% of them had never received an antihelmintic drug and 42% of horses had never been examined by a veterinarian. In relation to shoeing practices, all owners declared that their horses were periodically shod, every 15, 16–30 or more than 30 days (14%, 33% and 53% respectively). Within this sample, 48% were shod by the owner and 52% by a farrier. However, only 75% of horses were shod at the moment of inspection. Within the horses that were not shod (25%), 52% of them had a poor hoof quality. A significantly higher number of horses (75%) (*X*^2^ = 31.64; *p* = 0.000) were shod by owners in the Araucania region, while in the Metropolitana de Santiago region most horses (81%) were shod by a farrier. No significant difference (Wilcoxon rank sum test, *p* = 0.51) was found in the frequency of shoeing. Most owners reported that their horses were used for transporting their families (52%) and for carrying diverse types of loads. The diversity of products transported varied depending on the region where they were located. In the Araucania region, horses were mainly used for carrying agricultural products (58%) and wood for construction and fuel (42%). In the Metropolitana de Santiago region, horses were used for transporting potting soil (27%), agricultural products for sale in markets (27%), and rubble (8%). The average frequency of use was 3.25 days per week (SD = 1.65; range = 1–7) at an average of 3.87 hours per day (SD = 2.03; range = 1–12). No significant differences in the frequency of hours per day of work (Wilcoxon rank sum test, *p* = 0.58) and of days per week (Wilcoxon rank sum test, *p* = 0.11) were found between regions.

### 3.3. Interactions between Animal-Based Information and Indirect Indicators

The presence of skin lesions was not significantly associated with any of the following variables: poor body condition score (Fisher′s exact test, *p* = 0.06), inadequate conformation for draught activities (*X*^2^ = 0.37; *p* = 0.54), work frequency greater than 5 days/week (Fisher’s exact test, *p* = 0.13) or more than 6 hours/day (Fisher’s exact test, *p* = 0.10), nor individuals older than 15 years of age (Fisher′s exact test, *p* = 1.00). However, there was a tendency for horses to present lesions on the skin when in a poor body condition.

Inadequate body condition (found in 17% of the horses) was significantly associated (*X*^2^ = 4.86; *p* = 0.02) with the absence of deworming. However, no association (Fisher′s exact test, *p* = 0.14) was found between poor body condition and feeding less than two times per day, work frequency more than 5 days per week (Fisher′s exact test, *p* = 0.69) or more than 6 hours per day (Fisher′s exact test, *p* = 0.73), nor with individuals older than 15 years of age (Fisher′s exact test, *p* = 1.00).

Hoof abnormalities were significantly associated (*X*^2^ = 5.63; *p* = 0.01) with a low frequency of shoeing (over 30 days). In this study, no significant association (*X*^2^ = 0.05; *p* = 0.82) was found between an appropriate hoof condition and a history of the horse being shod by a farrier. There was no association between the presence of lameness and hoof abnormalities (Fisher′s exact test, *p* = 0.36).

Most horses had an alert attitude (97%), which was significantly associated with unlimited access to drinking water during the day, while they were not working. (Fisher′s exact test, *p* = 0.03).

### 3.4. Perceptions of Horses

Linguistic (semantic) analyses carried out showed that horse owners′ discourses were characterized by a high degree of subjectivity, with a high recurrence of terms such as “partner”, “like”, “friend”, “my life”, “all for me” or “family member” (e.g., “He is a family member, as a son”). Moreover, this subjectivity can be reflected in that all horses had a name given by the owner (e.g., Shakira or Gringo). In addition, 60% of the owners (63% from the Metropolitana de Santiago and 58% from the Araucania region) used possessive adjectives, such as "my horse is…” or “they are my…”, in their declarations.

Cluster analysis was used to identify related concepts or words according to their proximity in the owners′ answers. As shown in Table 7, both groups of owners tended to conceptualize their horses as either a friend, a family member, part of the home and/or a working tool. Furthermore, they recognized psychological attributes in their horses, such as the capacity for understanding or loyalty, and stated that they like having a horse. Some differences were observed between regions; five out of nine of the word clusters from the Metropolitana de Santiago region identified concepts that reflected the willingness of owners to care for their animals (e.g., “feed them”, “take care of them”) and indicated that horses were perceived mainly as a source of recreation for their owners (e.g., “hobby”, “toy”, “pets”, “favorite”). On the other hand, most word clusters (seven) from Araucania region identified a wider range of concepts that reflected the use of animals as the owners′ main source of subsistence (e.g., “transport”, “food”, “we eat”, “source”, “bread”, “plough”).

A Semantic Correlation Analysis among the most frequently used words by horse owners of the Metropolitana de Santiago region revealed a significant and very high correlation (*r* = 1.00; *p* < 0.05) between the following terms: “family” and “house”, “favorite” and “pet”, “distracts” and “take care of them”, “feed them” and “pet”, and “favorite” and “feed them”. Terms such as “feed them”, “take care of them”, “distracts”, “pet” and “favorite” were highly correlated with the term “like” (*r* = 0.7; *p <* 0.05) and the terms “livelihood” and “tool” were moderately correlated (*r* = 0.57; *p* < 0.05). 

In the Araucania region, analysis of semantic correlation showed a very high and significant correlation (*r* = 1.00; *p* < 0.05) between the terms: “provide” and “food”, “transport” and “like”, “apart” and “brother”, “transport” and “house”, and “bread” and “feeding”. A high correlation (*r* = 0.7; *p* < 0.05) was found between: “source” and “feeding”, “tool” and “food”, “income” and “ plough”, “family” and “apart”, “source” and “we eat”, “brother” and “family”, and “unique” and “understands”. 

Acording to the LSA cosine value, the terms used by the owners of the Metropolitana de Santiago region, such as “loyal” (cosine value 1.00) and “favorite” (cosine value 0.005), showed the closest proximity to the term "horse”. In the Araucania region, the words with the strongest proximity to the word “horses” were “family” (cosine value 0.99), “brother” (cosine value 0.98) and “friend” (cosine value 0.30). 

## 4. Discussion

Working equids contribute directly and indirectly to the livelihoods of the poorest communities around the world [4,32]. More specifically, they contribute to income-generating activities through the transport of goods, people, water, agricultural products, and building material. They also provide draught power for agriculture, among other activities [5,8,9,37,38].

In the present study, most working horses assessed had a good welfare status. This is contrary to reports from other studies that describe the welfare status of these animals as often poor [5,6,38,39,40]. This may be the result of labor demands by their owners and their relationship with people and their environment [41]. Additionally, welfare status may be affected by individualities (i.e., personality).

In this study, most owners showed a preference for using mares (61%), differing from previous studies where geldings were more frequent [9,10]. This could be due to owners′ considering the reproduction of their animals as an extra source of income through the sale of the offspring or as an asset, by keeping the newborns as replacement. Lanas et al. [11] reported a similar preference for mares in peri-urban areas of central and southern Chile.

Age is an important factor that may affect the welfare of working equids. McLeod [42] suggests that horses work better between 4–12 years of age. This is because horses reach their zootechnical maturity at approximately 4 years of age. In comparison, after 12 years of age, work, efficiency progressively decreases [42,43]. The average age of horses in this study was 8.7 years, which is similar to findings for other areas of Chile [9,10,11,42]. In addition, the majority of the horses (68%) included in this study were within the optimum age range, which as Sáez et al. [10] pointed out, has positive implications for the welfare of these animals.

A small percentage of horses examined in this study (17%) had an inadequate BCS, similar to the findings of Tadich et al. [9] in urban draught horses in southern Chile and contrary to those reported by Pritchard et al. [5], where the majority of working horses (70%) in Middle East and Central Asia had a BCS of 2 or less. It has been described that a low BCS can be caused by multiple factors, such as malnutrition, overwork, parasitism and diseases [7]. However, in this study, the only factor significantly associated with a poor BCS was the absence of deworming practices, found in 17% of all horses. Therefore, educational programs for owners focusing on good husbandry and healthcare practices should be implemented, with the purpose of improving owners′ understanding on how these practices can potentially affect the health and well-being of their animals.

Lameness, hoof abnormalities and poor hoof care are problems widely reported in working equines [6,9], mostly in urban or peri-urban areas [44]. In this study, hoof abnormalities were the most frequent problem, with no differences between the administrative regions studied. The majority of horses included in this study (53%) were shod infrequently, at intervals longer than 30 days. Additionally, there was a significant association between hoof abnormalities and shoeing intervals higher than 30 days. This low frequency of shoeing could be attributed to the lack of knowledge of owners about hoof balance and care and the lack of availability and accessibility of the service, taking into account the economic constraints of this population [3,45]. Furthermore, there was no association between adequate hoof conformation and horses being shod by a farrier, which may indicate deficiencies in farrier knowledge and skills, probably due to a lack of farrier training courses in the country [9]. Thus, farrier training courses on hoof care, aimed at local farriers and owners, should be implemented to increase horse owner awareness of the negative consequences that hoof abnormalities can bring for their animals′ welfare. This could be organized by local government agencies or non-governmental organizations in Chile.

Skin lesions have been reported as one of the most common afflictions found in working equids worldwide [5,6,9,10,38]. In this study, skin lesions were the second most frequently observed problem, especially in the Metropolitana de Santiago region. Most of these were simple excoriations, located mainly on harness related areas and in concordance with the patterns reported by other studies [9,44]. In our study, skin lesions tended to be associated with poor body condition, whereas age (individuals over 15 years of age) was not significantly associated with the presence of lesions, contrary to the findings of Burn et al. [6]. Likewise, the presence of skin lesions was not associated with an inadequate conformation of horses for draught activities (based on their AI and work frequency). Thus, other factors, probably extrinsic to the animal, could be associated with the occurrence of skin lesions, for example, ill-fitting harnesses, poor maintenance of equipment, or overload, all of which have been previously related to skin lesions in working equids [4,9].

Behavioral tests are considered an essential component in the welfare assessment of working equids. These tests indicate, to some extent, how the animal interacts with its environment [5] and can help to understand the quality of the human–equid relationship [7]. The high prevalence of an alert attitude and of positive (friendly) responses towards both the owner and the observer, in all tests in the two administrative regions evaluated, indicates the existence of a good human–animal relationship between owners and their animals and an appropriate handling of horses by owners. These results differ from previous studies, in which indifference, aggressiveness and avoidance were the most prevalent behaviors observed in working equids [4,5]. Several studies in the livestock industry have provided evidence on the relationship between the attitudes of a stockperson towards the animals and their behavioral responses and welfare [23,46]. For example, Hemsworth et al. [47] found that positive attitudes toward animals were associated with more positive interactions and that these positive interactions were negatively associated with the animals′ fear towards humans. Therefore, it could be possible that the owners assessed in this study have more positive attitudes towards their animals, which may be reflected in the high prevalence of the positive responses found. The discourse analysis of statements made by owners, focusing on how they conceptualize and describe their horses, showed similarities and differences, depending on the geographical location of their owners. Owners of both regions generally tended to see their animals as subjective individuals, capable of occupying a place in their lives as either a member of the family or as a friend. This indicates an affective relationship between the owners and their horses. Owners also conferred their animals’ mental abilities similar to those of humans (e.g., “He is the only one who understands me”), which can be interpreted as a clear tendency to anthropomorphize the behavior of their animals [48]. However, the recognition of these animals as a work tool (instrumental role) was also present in the discourse of owners, especially in owners from the Araucania region (e.g., “He is the one that provides my everyday bread, the food, my work tool”).

Generally, the emotionality (affection for animals) and instrumentality (e.g., economic or utilitarian considerations) with which animals are perceived are often seen as opposite to each other [17,18]. In this sense, the emotional aspect of the human–animal relationship has often been linked to the keeping of companion animals [49], whereas keeping farm animals has been mainly associated to financial or utilitarian purposes, especially in the poorest communities worldwide. However, the results from this study suggest that affective and instrumental perceptions can coexist. Coincidentally, Schuurman [18] showed that conceptions of horses, particularly of equine welfare, consist of a combination of emotional and instrumental relations. Other authors have also reported that instrumental and affective components within livestock production can, and do, co-exist. In other words, animals can be thought of as simultaneously a friend and source of food [50,51].

How people relate to animals cannot be isolated from the cultural contexts in which they are embedded [51]. In the Metropolitana de Santiago region, owners′ descriptions of their horses indicated predominantly affective-type ascriptions. Based on a correlations analysis, the results indicate that the owners of this region showed a certain predilection for this species and tend to perceive their horse, primarily, as a pet, even in some cases as a friend. Moreover, they recognized certain needs of their animals and demonstrated a clear intention in taking care of them (e.g., “They are my favorite pets, I like to feed them well”), most likely to keep their animals as healthy as possible. This could be interpreted as the owners having an empathetic understanding of their horses as individuals (in some cases, they are considered companion animals or pets) that feel and have needs. In the Araucania region, owners′ discourse emphasized the perception of their horse as a family member. This indicates, based on the LSA results, that there may be an affective owner–horse relationship, due to the owners′ perception of kinship and closeness with their animals. The owner′s discourse also highlighted the instrumental role that their horse plays for the family′s subsistence, exalting a feeling of gratitude towards the animal′s labor. One possible explanation for this ambivalence could be that owners from the Araucania region are closer to an ethnic-rural background than those from the Metropolitana de Santiago region. The Mapuche ethnicity, which is concentrated in the Araucania region [14], has been characterized as a culture that historically conceived the horse (a species introduced by the Spanish) as an animal of burden and transport [52]. Therefore, it might be expected that these owners have acquired a utilitarian vision of these animals through family and cultural inheritance. On the other hand, owners from the Metropolitana de Santiago region do not see the horse exclusively as a working instrument but also as a companion animal. In fact, results of the LSA suggest that there is predilection towards horses, rather than a sense of labor in keeping these animals, and that owners from this region also recognize human psychological characteristics in their animals.

It is important to note that most owners stated that they enjoyed keeping these animals, which could indicate that many of them chose this occupation voluntarily, rather than simply continuing a family activity. These perceptions, added to the good welfare status found in most of the horses evaluated in the study, illustrate that working horse owners in Chile, generally, do not mistreat or exploit their animals.

This study may be limited by owners′ bias when asked about perceptions regarding their horses. Survey respondents may have tried to anticipate what they thought researchers wished to hear. This is especially likely to occur within this population, considering that working horses are constantly in the public eye [53,54,55]. Given that an owner′s appreciation of his/her horse(s) was determined through only one question, future studies could include the use of validated attachment scales and attitudes towards animals.

## 5. Conclusions

The low prevalence of health problems and negative behavior responses found in horses of the two administrative regions evaluated indicate a good welfare status for the majority of horses in this study. The main problems observed in the assessed animals were hoof abnormalities and skin lesions. A possible explanation for these observations is a lack of understanding of basic husbandry practices, which may be improved through the implementation of educational programs for owners and local farriers. There were significant differences between the two regions in the presence of lesions on horses and the person responsible for shoeing management. This study provides preliminary information about owners′ relationships with their animals and both affective and instrumental perceptions of working horses were observed. While the instrumental perception was more common in the Araucania region, the affective perception was widely shared by both groups of owners. This indicates that working horses are considered part of the family or a friend and suggests an affective relationship of owners with their animals.

## Figures and Tables

**Table 1:** Description of the direct health parameters applied.

Welfare Indicators	Categorization	Description
Skin lesion	Present/absent	Wounds of any size and severity were recorded according to their location. Lesions at labial commissures of the mouth were also included.
Body condition score	Adequate/inadequate	Assessed on a five-point scale from 1 (emaciated) to 5 (obese) including half scores [6,32]. Scores of 3, 3.5 and 4 were considered adequate.
Hoof health	Adequate/inadequate	Quality, shape and conformation of hoofs were assessed. The hooves were considered adequate if these were round and smooth, had no cracks or sections missing, and did not show defects of the hoof capsule [31,33].
Coat and skin condition	Adequate/inadequate	The coat and skin condition was recorded adequate if the hair coat was uniform, with a general healthy aspect (shiny), without dryness or dirt (mud or feces) or presence of ectoparasites of any species (in hair or skin) [5,33].
Gait abnormalities	Present/absent	Assessed by observation of the horse while walking in a straight line for approximately 20 meters. The observer assessed presence of lameness, uneven stride, reluctance to put weight on one or more limbs, uneven head-nodding or hip movement [31].

**Table 2:** Description of the direct behavioral observations applied.

Indicator	Categorization	Description
General attitude	Alert/apathetic or depressed	The horse was observed (only by observer) from a distance of 3 to 5 meters for 60 seconds [4]. The horse’s response was categorized as: Alert: when the animal was attentive and responds to the different stimuli of the environment (eyes wide open, active movement of the ears, head, tail and/or skin to keep away flies) [4]. Apathetic or depressed when it showed decreased responses to the environmental stimuli (head lowered, eyes half closed, complete or partial cessation of tail and skin movements to avoid insects, reduced ear movement) [4,31]. Apathetic and depressed were combined in the current study based on criteria of Burn et al. [31].
Approximation test	Indifference/friendliness/ avoidance/aggressiveness	The observer approached at an angle of approximately 20° to the sagittal plane of the animal’s body and stopped at a distance of 30 cm from the head of the horse [4]. The observer recorded the horse’s response at the moment that he stopped. The owner was instructed to perform exactly the same procedure and then the observer recorded the animal’s response [4]. Responses were recorded as: Indifference: Immobile and relaxed without attempts to approach or move away from the observer/owner, depressed or relaxed body position and facial expression (with or without the ears moving, relaxed lips, possibly eyes half closed) [4]. Friendliness: Movement of the head toward the observer/owner, with relaxed face and normally open eyes, ears turned forward, no wrinkling around the mouth or nostrils [4]. Avoidance: The horse is immobile with a tense body position and facial expression (head up, eyes wide open and lips held tight) or the animal turning the head or attempts to move away from observer/owner [4]. Aggressiveness: The horse attempts to kick or bite, eyes fully opened and head oriented toward observer/owner, nostrils are dilated with or without wrinkles around the mouth, may paw or stomp the ground [4].
Walk down side	Indifference/friendliness/ avoidance/aggressiveness	The observer walked alongside the horse toward its rear and back again, maintaining a distance of 30 cm from its body, then the observer recorded the horse’s response [4]. The owner was instructed to perform the same procedure. The horse′s response was categorized exactly as in the approximation test [4].
Chin contact	Accepts/avoids	The observer slowly placed their hand under the animal’s chin and assessing if the horse accepted or avoided the contact [31]. The owner was instructed to perform the same procedure. The horse′s response was categorized as accepts or avoids the contact [31].
Allows to pick up a limb	Accepts/avoids	The observer assessed if the horse resisted or not the lifting up of their left front limb. The owner was instructed to perform the same procedure.

**Table 3:** Description of the indirect indicators (resources and management) applied.

Welfare Indicators	Categorization	Description
*1. Feeding practices*		
Frequency of feeding	Once a day/twice a day/three or more per day	The owner was asked how many times per day he/she supplied water to their horse.
Water availability	*Ad libitum/not ad libitum*	The owner was asked if their horse had water available *ad libitum* when not working.
*2. Working practices*		
Frequency of use per day	Days per week	The owner was asked about how many days per week he/she uses the horse for work
Frequency of use per week	Hours per day	The owner was asked how many hours per day he/she uses the horse for work.
Work type	Type of load	The owner was asked about the activities in which he/she uses the horse.
*3. Shoeing practices*		
Frequency of shoeing	Every 15/between 16–30/>30 days	The owner was asked about the frequency that his/her horse is shod.
Responsible person	Farrier/owner	The owner was asked about the main person responsible of the shoeing of the horse.
*4. Preventive management*		
Deworming	Never/<6 month/>6 month	The owner was asked when was the last time his/her horse was dewormed. The response was categorized as never; less than 6 months ago; or more than 6 months ago.
*5. Veterinary consultation*	Never/<1 year/>1 year	The owner was asked about the last time his/her horse was examined by a veterinarian. The response was categorized as never (if the horse has never been examined by a veterinarian); less than a year ago; or over a year ago.

**Table 4:** General characteristics of urban working horses (*n* = 100) assessed from the Metropolitana de Santiago (*n* = 48) and Araucania (*n* = 52) regions in Chile. Results are expressed as average, standard deviation (SD), range, percentage (%) and number (*n*).

Descriptor	Metropolitana de Santiago (*n* = 48)	Araucanía (*n* = 52)	Total (*n* = 100)
Average age (years (SD))	8.1 (3.7) ^a^	9.2 (5.1) ^a^	8.7 (4.5)
Age range	2–15	2.5–25	2–25
Estimated live weight average (kg (SD))	388 (81.4) ^a^	436 (76.1) ^a^	413 (82)
Anamorphosic index adequacy for draught activities (% (*n*))	23 (11) ^a^	37 (19) ^a^	30 (30)
Geldings (% (*n*))	10 (5) ^a^	46 (24) ^b^	29 (29)
Stallions (% (*n*))	19 (9) ^a^	2 (1) ^b^	10 (10)
Mares (% (*n*))	71 (34) ^a^	52 (27) ^a^	61 (61)

^a, b^ Different letters denote significant differences (p < 0.05) between administrative regions.

**Table 5:** Descriptive statistics of health indicators of 100 draught horses assessed from the Metropolitana de Santiago (*n* = 48) and Araucania regions (*n* = 52) in Chile, expressed in number (*n*) and percentage (%) within each region. Significant differences between regions are also shown.

Indicators	Metropolitana de Santiago	Araucania	Total	*p-*Value
	*n* (%)	*n* (%)	*n* (%)	
Inadequate body condition score	7 (15)	10 (19)	17 (17)	0.53
Presence of body lesions (skin)	30 (63)	17 (33)	47 (47)	<0.05
Lesions at the labial commissures	3 (6)	0	3 (3)	0.10
Head/neck	17 (35)	3 (6)	20 (20)	<0.001
Breast/shoulder	9 (19)	6 (12)	15 (15)	0.31
Thorax/abdomen	13 (27)	11 (21)	24 (24)	0.48
Hindquarters/tail base	9 (19)	4 (8)	13 (13)	0.13
Forelegs/hindlegs	10 (21)	4 (8)	14 (14)	0.08
Abnormal coat and skin	8 (17)	6 (12)	14 (14)	0.46
Abnormalities of hoof	25 (52)	28 (54)	53 (53)	0.85
Abnormal gait/lameness	9 (19)	4 (8)	13 (13)	0.13

**Table 6:** Descriptive statistics of behavioral indicators of 100 draught horses assessed from the Metropolitana de Santiago (*n* = 48) and Araucania regions (*n* = 52) in Chile, expressed in number (*n*) and percentage (%) within each region. Significant differences between regions are also shown.

Indicators	Metropolitana de Santiago	Araucania	Total	*p-*Value
	*n* (%)	*n* (%)	*n* (%)	
General attitude				
Alert	46 (96)	51 (98)	97 (97)	0.60
Apathetic/depressed	2 (4)	1 (2)	3 (3)	0.60
Response to observer approach				
Indifference	11 (23)	1 (2)	12 (12)	<0.001
Friendly	30 (63)	44 (85)	74 (74)	<0.01
Avoidance	3 (6)	6 (12)	9 (9)	0.49
Aggression	4 (8)	1 (2)	5 (5)	0.19
Response to owner approach				
Indifference	8 (17)	2 (4)	10 (10)	<0.05
Friendly	32 (67)	46 (88)	78 (78)	<0.01
Avoidance	5 (10)	3 (6)	8 (8)	0.47
Aggression	3 (6)	1 (2)	4 (4)	0.34
Response to observer walking down side				
Indifference	16 (33)	5 (10)	21 (21)	<0.01
Friendly	26 (54)	38 (73)	64 (64)	<0.05
Avoidance	2 (4)	6 (12)	8 (8)	0.27
Aggression	4 (8)	3 (6)	7 (7)	0.70
Response to owner walking down side				
Indifference	9 (19)	2 (4)	11 (11)	<0.05
Friendly	32 (67)	44 (85)	76 (76)	<0.05
Avoidance	3 (6)	5 (10)	8 (8)	0.71
Aggression	4 (8)	1 (2)	5 (5)	0.19
Response to observer making chin contact				
Acceptance	36 (75)	41 (79)	77 (77)	0.64
Avoidance	12 (25)	11 (21)	23 (23)	0.64
Response to owner making chin contact				
Acceptance	39 (81)	43 (83)	82 (82)	0.85
Avoidance	9 (19)	9 (17)	18 (18)	0.85
Response to observer picking up a limb				
Acceptance	44 (92)	49 (94)	93 (93)	0.70
Avoidance	4 (8)	3 (6)	7 (7)	0.70
Response to owner picking up a limb				
Acceptance	45 (94)	50 (96)	95 (95)	0.66
Avoidance	3 (6)	2 (4)	5 (5)	0.66

**Table 7:** Word clusters originated from working horse owners’ answers to the open question “What does your working horse mean or represent for you?”, according to the location of origin, either Metropolitana de Santiago region (*n* = 48) or Araucania region (*n* = 52).

Clusters	Metropolitana de Santiago Region	Araucania Region
cluster 1	friend, animals, horses	son, horse, feeding
cluster 2	tools, animals, feed them	house, like, transport
cluster 3	hobby, toy, time	friend, rescuer, feeding
cluster 4	loyal, horse, feed them	food, tool, bread
cluster 5	feed them, like, pets	unique, understands, life
cluster 6	horse, feel, feed them	we eat, eat, source
cluster 7	tool, animal, livelihood	foods, provides, feeding
cluster 8	like, feed them, take care of them	friend, apart, horse
cluster 9	friend, favorite, home	source, plough, feeding
